# Supplementary material for: Rational design of Al2O3/2D perovskite heterostructure dielectric for high performance MoS2 phototransistors
Source: Nat Commun. 2020 Aug 26;11:4266. doi: 10.1038/s41467-020-18100-9 (PMC7450060; doi:10.1038/s41467-020-18100-9)
Supplement: Supplementary file 1 — Supplementary information [file 41467_2020_18100_MOESM1_ESM.pdf]

Supplementary Information

Rational Design of Al_2O_3/2D Perovskite Heterostructure Dielectric for High Performance MoS_2 Phototransistors

Jiayang Jiang[1], Xuming Zou[1]*, Yawei Lv[1], Yuan Liu[1], Weiting Xu[1], Quanyang Tao[1], Yang Chai[3], Lei Liao[1,2]*

[1]*Key Laboratory for Micro/Nano Optoelectronic Devices of Ministry of Education & Hunan Provincial Key Laboratory of Low-Dimensional Structural Physics and Devices, School of Physics and Electronics, Hunan University, Changsha 410082, China.*

[2]*State Key Laboratory for Chemo/Biosensing and Chemometrics, School of Physics and Electronics, Hunan University, Changsha 410082, China.*

[3]*Department of Applied Physics, The Hong Kong Polytechnic University, Hong Kong 999077, China.*

*Corresponding email: zouxuming@hnu.edu.cn; liaolei@hnu.edu.cn

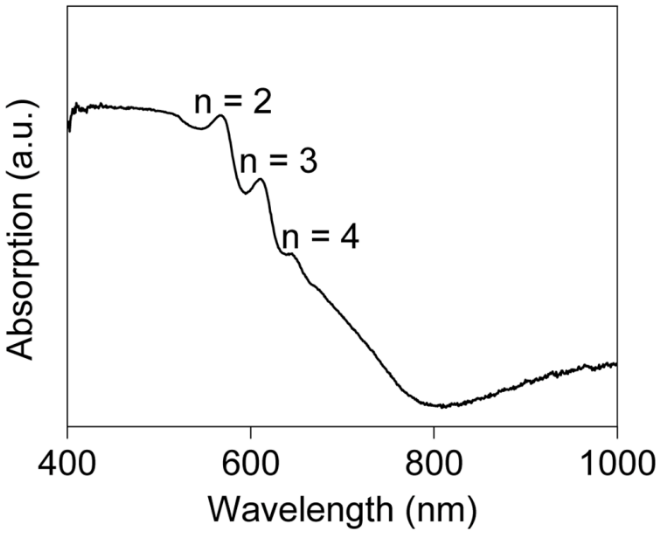

Supplementary Figure 1. Absorption spectrum of the 2D perovskite $(PEA)_2(MA)_2Pb_3I_{10}$ (n=3).

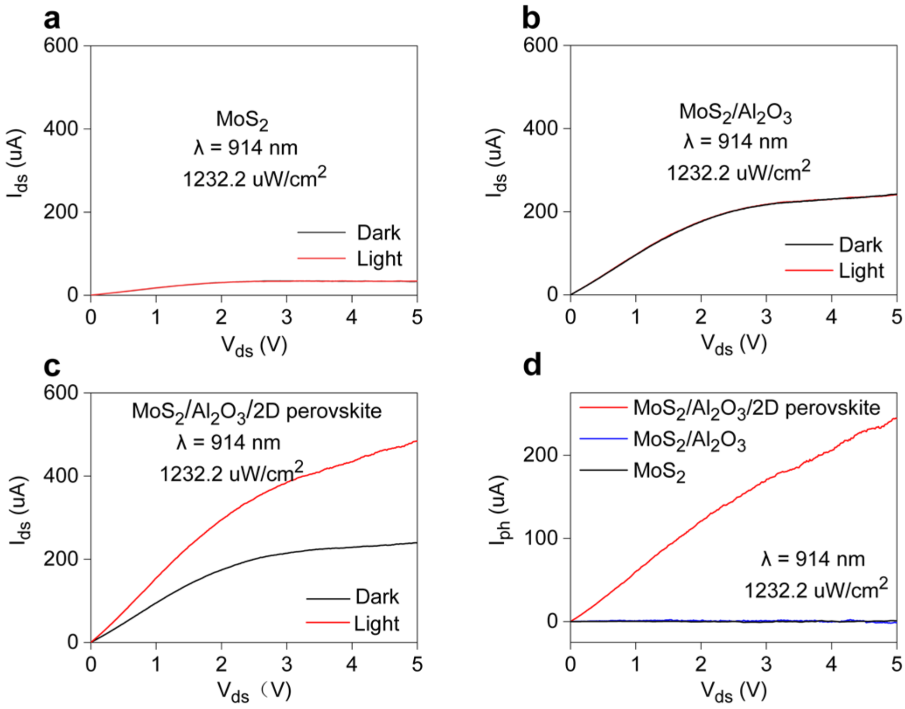

Supplementary Figure 2. Photoresponse of the photodetectors upon 914 nm illumination based on the same MoS_2 flake. The I_{ds}-V_{ds} curves of the photodetectors measured with and without illumination based on different device architectures of **a** bare MoS_2, **b** MoS_2/Al_2O_3, and **c** MoS_2/Al_2O_3/2D perovskite (n = 3). **d** The corresponding photocurrent of the devices.

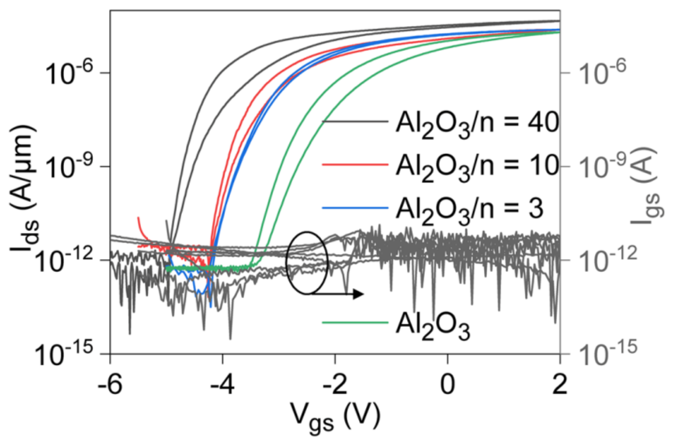

Supplementary Figure 3. Gate leakage curves of MoS_2 transistors with different gate dielectrics.

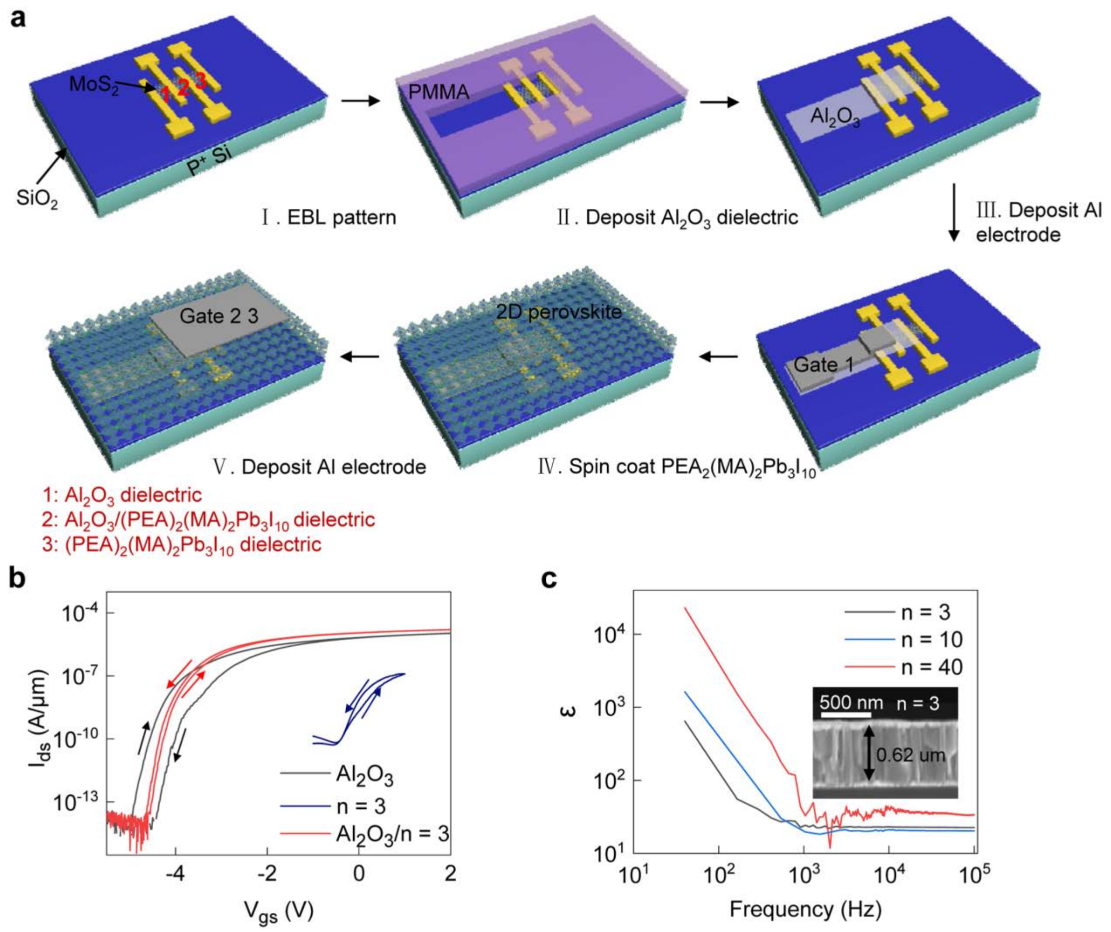

Supplementary Figure 4. The fabrication procedure and characterization of MoS_2 phototransistors with three types of dielectrics. a Schematic diagrams illustrate the preparation process of the devices. **b** Transfer characteristic curves of MoS_2 phototransistors with Al_2O_3, $(PEA)_2(MA)_2Pb_3I_{10}$, and $Al_2O_3/(PEA)_2(MA)_2Pb_3I_{10}$ dielectrics, respectively. **c** The extracted dielectric constant of 2D perovskites based on Au/2D perovskite/Au parallel plate capacitor. The inset gives the corresponding cross-sectional SEM image.

Here, we fabricate MoS_2 phototransistors with different dielectric to compare the dielectric properties. In order to avoid the error caused by different MoS_2 flakes, the phototransistors are fabricated with the same MoS_2 flake. Briefly, the conductive channel 1, 2, and 3 were firstly defined on the same MoS_2 flake. Then, a 9 nm thick of

Al_2O_3 layer was patterned onto channel 1 and 2 using EBL and ALD. 20 nm Al electrode was patterned on the channel 1 after the deposition of Al_2O_3 layer. Subsequently, $(PEA)_2(MA)_2Pb_3I_{10}$ film was deposited by one-step spin coating method. Finally, 20 nm Al electrode was formed on top of channel 2 and 3 by thermal evaporation through a shadow mask. In addition, we fabricate the Au/2D perovskite/Au parallel plate capacitor to estimate the dielectric constant (ε) of 2D perovskites. Here, a giant dielectric constant phenomenon in 2D perovskite at low frequency is observed, which is probably caused by intrinsically polarizability of perovskite[1]. In typical series capacitance geometry, the total capacitance of Al_2O_3/2D perovskite dielectric is given by:

$$C_{\mathrm{Al}_2O_3/\mathrm{2D\ perovskite}} = 1/(1/C_{\mathrm{Al}_2O_3} + 1/C_{\mathrm{2D\ perovskite}}) \approx C_{\mathrm{Al}_2O_3} \quad (1)$$

where $C_{Al2O3/\mathrm{2D\ perovskite}}$ is the Al_2O_3/2D perovskite capacitance, C_{Al2O3} is the Al_2O_3 capacitance, and $C_{\mathrm{2D\ perovskite}}$ is the 2D perovskite capacitance. Based on this model, the key performance parameters of the devices are extracted (Supplementary Table 1). In comparison with Al_2O_3 and Al_2O_3/2D perovskite dielectrics, the device with 2D perovskite dielectric exhibits much lower electrical performance. It is well known that the interface quality plays a crucial role in the carriers transport of 2D semiconductor devices[2]. This result indicates the severe carrier scattering involving surface roughness and Columbic impurity scattering at MoS_2/2D perovskite interface[3].

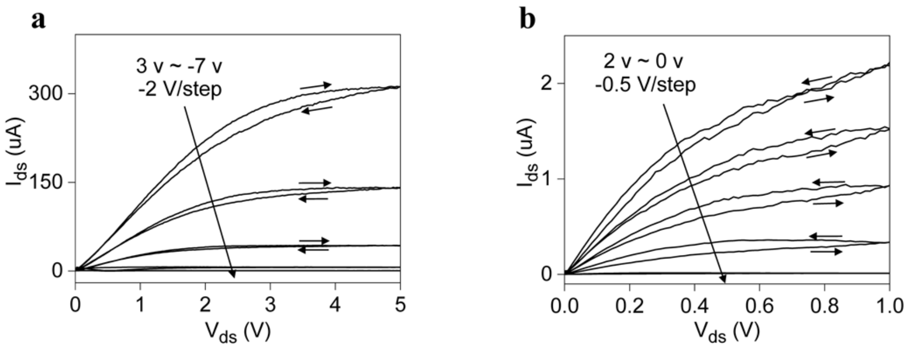

Supplementary Figure 5. Output characteristics of MoS_2 transistors. The dual sweep output curves of MoS_2 transistors with **a** single Al_2O_3 dielectric and **b** single $(PEA)_2(MA)_2Pb_3I_{10}$ dielectric.

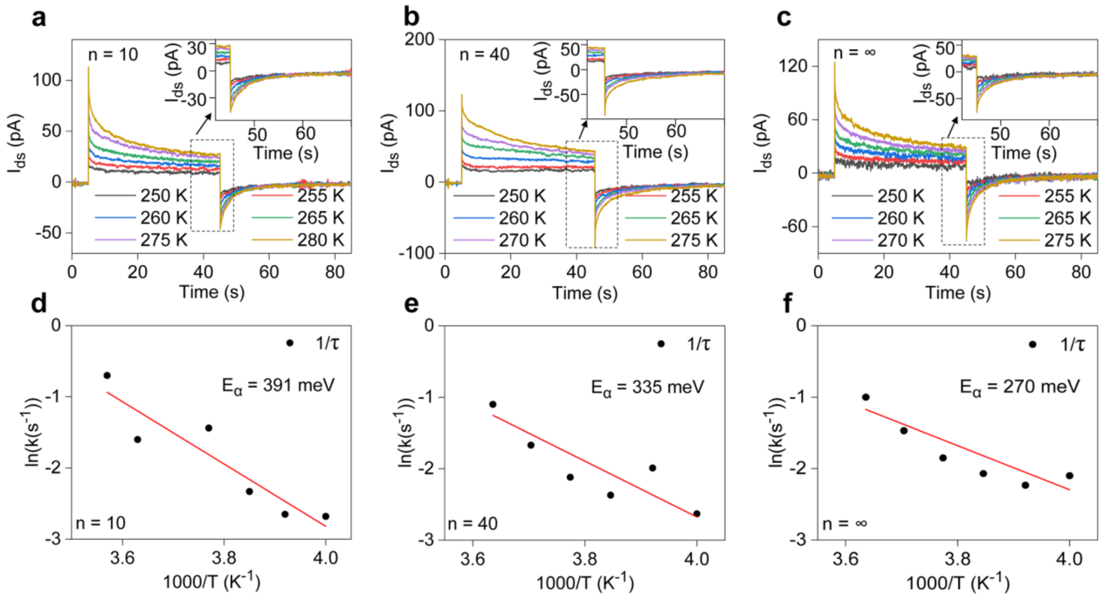

Supplementary Figure 6. Measured ionic migration in 2D perovskites based on vertically stacked Au/2D perovskite/Au devices. Temporal response curves for Au/2D perovskite/Au device measured under dark at **a** n = 10, **b** n = 40, and **c** n = ∞. Arrhenius plot of the ion decay rate *1/τ* at **d** n = 10, **e** n = 40, and **f** n = ∞. The solid line represents the fitting result.

The dynamic of ionic motion is reflected by the negative current decay. Accordingly, the decay curve of the negative current can be fitted with the biexponential function:

$$I(t) = I_0 + (I_e - I_1)\exp(-\frac{t-t_0}{t_e}) + (I_1 - I_0)\exp(-\frac{t-t_0-\delta}{t})$$

Where I_0 (essentially zero) denotes the constant total current of the Au/2D perovskite/Au device under equilibrium conditions without the applied external bias; t_0 denotes the starting point right after the removal of applied external bias; τ refers to the time constant of ionic diffusion and restoration; I_1 denotes the instant current right after the removal of the external bias at $t_0+\delta$; τ_e and I_e refer to the equipment response

(i.e., RC delay). The τ value can be extracted by fitting the current decay curve. Since the decay rate, $s = 1/\tau$, represents the ionic transport dynamics, we can extract E_α value by fitting temperature-dependent s value with Arrhenius equation:

$$\ln(s) = \mathrm{C} - \frac{E_a}{K_B T}$$

where C is a constant, and k_B is Boltzmann's constant.

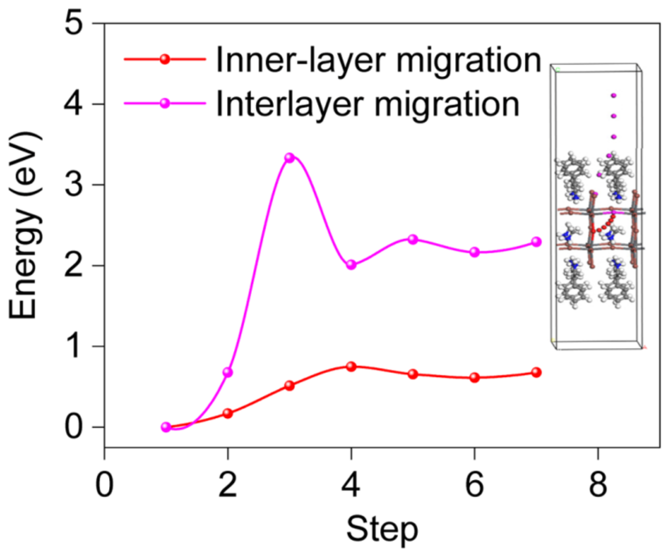

Supplementary Figure 7. Calculated energy barriers along the V_I migration path shown in the inserted plot. The red and pink paths denote the inner-layer and interlayer V_I diffusion in 2D perovskite crystal (n = 1), respectively.

An open-source package QUANTUM ESPRESSO is employed to perform the calculation of energy barriers and reaction pathway using the Nudged Elastic Band (NEB) method [10.1088/0953-8984/21/39/395502] [10.1088/1361-648X/aa8f79]. The number of ionic plus electronic steps and images within each path are 50 and 7. The steepest descent optimization scheme is adopted and the climbing image scheme is "auto". In the self-consistent electronic ground-state calculations, the Perdew-Burke-Ernzerhof (PBE) exchange-correlation functional with the ultrasoft pseudopotential is adopted and the plane-wave energy cutoff is 46 Ry. In Fig. S8, the supercell is chosen as 2 × 2 × 1 of the original cell. The calculated energy barrier for interlayer diffusion of V_I is 2.7 eV, much larger than that of 0.7 eV for inner-layer diffusion, indicating the distinct interlayer migration restraining effect of the organic ammonium layers.

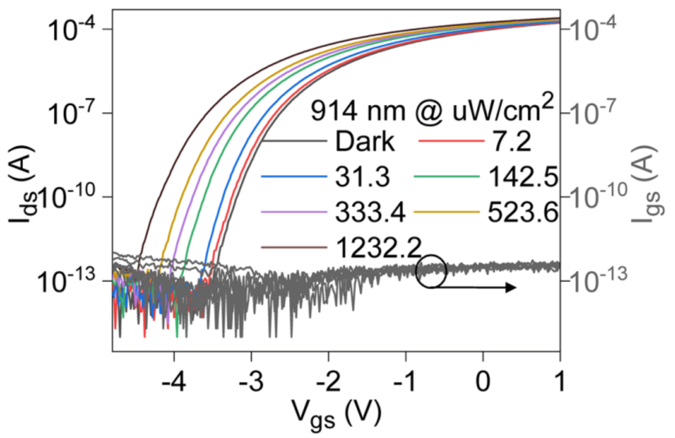

Supplementary Figure 8. Gate leakage curves of MoS_2 phototransistor under different laser power density.

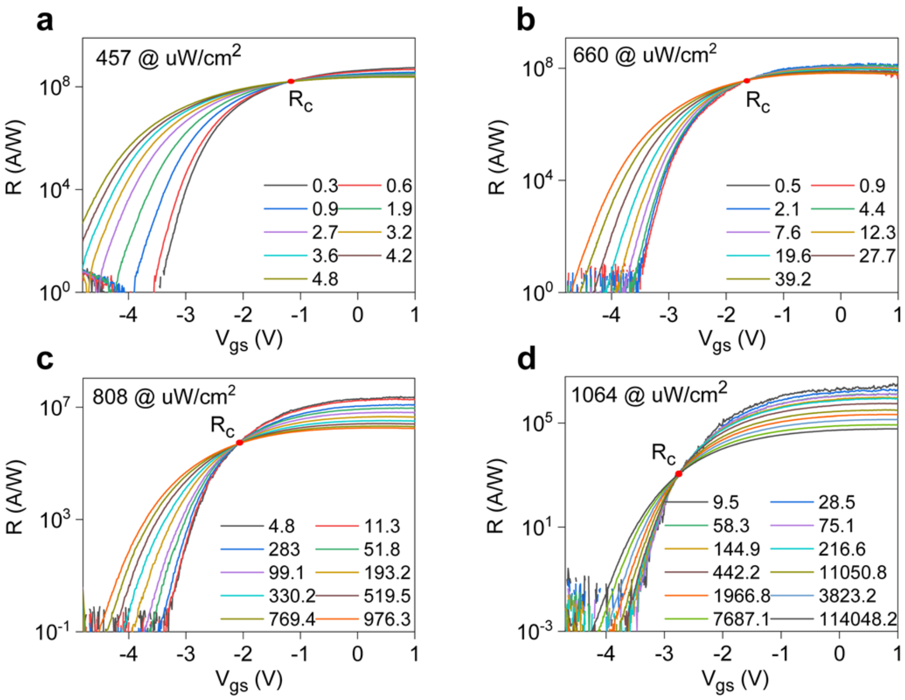

Supplementary Figure 9. Photoresponsivity characteristics of MoS_2 phototransistor with Al_2O_3/$(PEA)_2(MA)_2Pb_3I_{10}$ (n = 3) dielectric. Gate voltage and P_{light} dependent photoresponsivity under **a** 457 nm, **b** 660 nm, **c** 808 nm and **d** 1064 nm illumination.

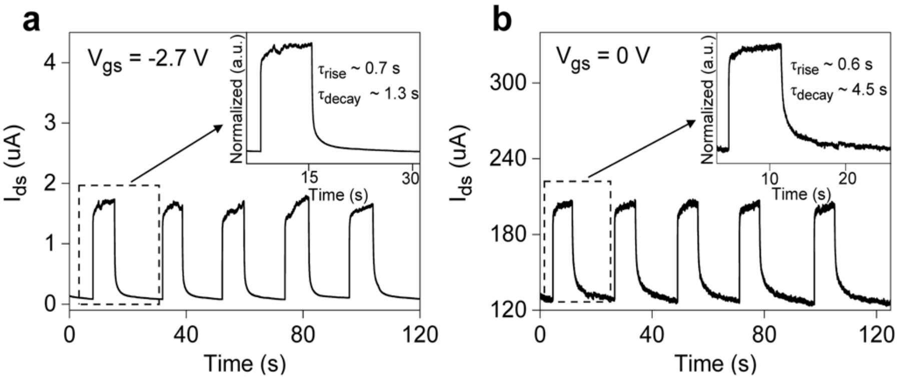

Supplementary Figure 10. Transient properties of the MoS_2 phototransistor upon 914 nm illumination. The rise and decay time of the phototransistor at **a** V_{gs} = -2.7 V and **b** V_{gs} = 0 V.

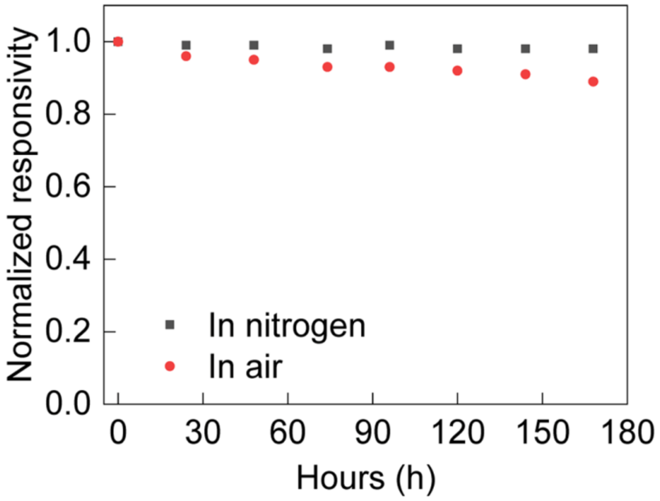

Supplementary Figure 11. Stability measurements of the phototransistor with Al_2O_3/2D perovskite dielectric at P_{light} = 1 mW/cm^2 and λ = 914 nm.

Supplementary Table 1. Comparison in device performance with different dielectrics.

Dielectric structure	μ_F/μ_B ($cm^2/V \cdot s$)	SS_F/SS_B (mV/dec)	V_{thF}/V_{thB} (V)	*Hysteresis* (V)	I_{on} (A/μm)	I_{on}/I_{off}
Al_2O_3	19.2/21.3	93/99	-3.2/-2.5	0.7	1.1×10^{-5}	2.8×10^{8}
$(PEA)_2(MA)_2Pb_3I_{10}$	1.2/0.8	220/182	0.3/0.1	0.2	1.3×10^{-7}	1.9×10^{3}
$Al_2O_3/(PEA)_2(MA)_2Pb_3I_{10}$	20.4/20.5	82/80	-3.2/-3.3	0.1	1.5×10^{-5}	4.8×10^{8}

F: Forward scan B: Backward scan

Supplementary References

1. Juarez-Perez, E. J. *et al.* Photoinduced giant dielectric constant in lead halide perovskite solar cells. *J. Phys. Chem. Lett.* **5**, 2390-2394 (2014).
2. Fiori, G. *et al.* Electronics based on two-dimensional materials. *Nat. Nanotechnol.* **9**, 768-779 (2014).
3. Hirai, H., Tsuchiya, H., Kamakura, Y., Mori, N. & Ogawa, M. Electron mobility calculation for graphene on substrates. *J. Appl. Phys.* **116**, 083703 (2014).
